# Observational Studies on Evaluating the Safety and Adverse Effects of Traditional Chinese Medicine

**DOI:** 10.1155/2013/697893

**Published:** 2013-09-14

**Authors:** Jung-Nein Lai, Jin-Ling Tang, Jung-Der Wang

**Affiliations:** ^1^Institute of Traditional Medicine, School of Medicine, National Yang-Ming University, Taipei City 112, Taiwan; ^2^Department of Chinese Medicine, Taipei City Hospital, Yangming Branch, Taipei City 111, Taiwan; ^3^Center for Evidence Based Medicine, Peking University Health Science Centre, Peking University, Beijing 100871, China; ^4^Division of Epidemiology, School of Public Health and Primary Care, The Chinese University of Hong Kong, Hong Kong; ^5^Department of Public Health, College of Medicine, National Cheng Kung University, Tainan City 701, Taiwan

## Abstract

*Background*. This study aims to share our experiences when carrying out observational studies of traditional Chinese medicine (TCM). *Methods*. We have proactively monitored the safety profiles of *Duhuo Jisheng Tang (DJT)*, *Suan Zao Ren Tang (SZRT)*, and TMN-1. A list of adverse events (AEs), complete blood counts, and liver and kidney function tests were obtained from the participants during their scheduled hospital visits. Retrospective observational studies were conducted based on the reimbursement database of the National Health Insurance system, Taiwan, to explore the relationship between the use of TCM that have been adulterated by aristolochic acid and the risk from both nephrotoxins and carcinogens. *Results*. A total of 221, 287, and 203 AEs were detected after *SZRT*, *DJT*, and TMN-1 had been taken, respectively. Dizziness, headache, stomach ache, and diarrhea were judged to be probably related to *SZRT* treatment. Retrospective observational studies found an association between the consumption of aristolochic acid-containing Chinese formulae such as *Mu Tong* and an increased risk of CKD, ESRD, and urinary tract cancer. *Conclusion*. *Prospective and retrospective* observational studies seem to have specific advantages when investigating the safety and adverse effects of TCM therapies, as well as possibly other alternative/complementary therapies.

## 1. Introduction

Traditional Chinese medicine (TCM), a long and widely used form of medical care in ethnic Chinese communities and nearby regions, has recently been adopted by other ethnic groups worldwide [1–5]. Most indications and contraindications of TCM therapies currently in the market are solely based on documentation found in ancient books [6] associated with a traditional belief that these herbs and/or their combinations are usually safe. Randomized controlled trials (RCTs) are recommended to evaluate the intended effects of TCM therapies because these are the most convincing design for controlling bias and potential confounding factors during comparative studies of clinical interventions [7–10]. While modern medicine has generally developed from physiology and biochemistry and the mechanisms of action of such drugs are understood at cellular and molecular levels, the therapeutic principles of TCM usually take a holistic view involving activating systems, improving system connection, and enhancing human disease resistance [11]. TCM doctors usually prescribe herbs tailored to the patient's symptoms/signs and constitution, which often requires some modification of the ancient Chinese formulae. As a result, herbal formulae that consist of modified prescriptions of herbs continue to appear and are also prescribed without any systematic evaluation [1]. Thus there remains a big question in TCM research as to how to evaluate TCM prescriptions; this is because they are highly individualized and differ from patient to patient. Thus, one possibility is the evaluation of many different herbal formulae during the same trial or another possibility is to adopt an approach that differs from that used when evaluating purified-compound medicines. Without prior pilot studies to generate safety and efficacy data, it is not surprising that very few TCM therapies have undergone high-quality clinical trials and have been proven to effectively treat diseases or symptoms [9, 12, 13]. Furthermore, because there are considerable variations in the quality of TCM trials, negative results from RCTs cannot be used as sufficient evidence for the absence of efficacy, especially when these TCM remedies have already been on the market or have been approved by regulatory agencies. Ephedrine and artemisinin are currently used in western medicine for treating asthma and malaria, respectively, and have a long history of use as Chinese herbal remedies. These examples indicate that there the clinical effects of TCM therapies show a positive association between the active ingredients used in modern therapies [14, 15] and the traditional use of the plants from which they are derived. Thus it should be recognized that, for some, the effects of traditional Chinese medicines when treating symptoms are as effective as conventional western medicines, but this also gives them the same potential to have harmful side effects [16]. Furthermore, adulteration, inappropriate formulation, and a lack of understanding of Chinese herbs and their interactions have led to adverse reactions that are sometimes life threatening or lethal. Thus, the safety concerns normally applied to conventional medicines equally apply to traditional Chinese medicines.

**Figure 1:**
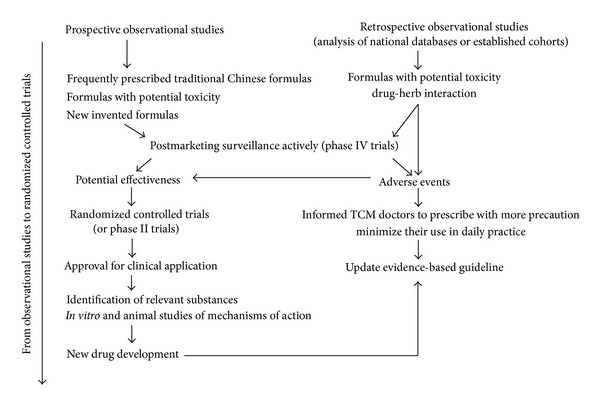
Surveillance of safety and adverse effects of traditional Chinese medicine.

Unlike efficacy data, safety information obtained even from case series [17] has direct and immediate applications to patient care. If a treatment is proved to be misused or involved in oversight during the course of clinical care, then stopping their use immediately will protect lives and save resources. Aristolochic acid nephropathy (AA nephropathy), which has been observed in patients taking *Mu Tong* and *Fangchi *(traditional Chinese formulae that have been adulterated by aristolochic acid), is a good example. If the causation between a side effect and a TCM therapy can be corroborated, updating contraindication of the treatment will benefit future TCM users. As Chinese herbal medicines are used in humans all the time, continuous surveillance of patient safety while taking TCM treatments, which can be likened to postmarket surveillance, should be a convenient and powerful way to detect any potential harm caused by a TCM therapy. In this paper, we share our experiences conducting prospective observational clinical studies that use active surveillance of the safety profiles of various TCM therapies (Figure 1, left column). In addition, a retrospective observational study was also conducted involving the passive surveillance of the safety profile of Chinese formulae that are known to have been adulterated with *Aristolochia* spp. These approaches we believe, when used to assess the safety of Chinese herbal therapies, may have wider applications.

## 2. Materials and Methods

### 2.1. Study Design 1: Active Surveillance of Safety Profiles

An effective way of detecting TCM safety hazards is through active surveillance of a well-defined group of people who are taking the medication, who are then followed using the guidelines of GCP (good clinical practice) [18]. First we organized at the beginning of study design an expert focus group composed of practicing TCM doctors to determine if they had noticed any adverse events (AEs) associated with the treatment of subjects with a specific health condition. Then, these reported potential AEs were included in our list for surveillance with the AEs most frequently reported for conventional medicine by the National Reporting System; these consisted of abdominal fullness, diarrhea, vomiting, nausea, urticaria, itching, purpura, jaundice, skin vesicles (or local reddish swelling), edema, hypotension, bradycardia, dyspnea, fever, muscle cramps, and sleepiness. In addition, all subjects were required to take objective laboratory tests in order to detect any abnormal findings with respect to liver function, kidney function and blood counts, both at baseline and after TCM therapy. During the active safety surveillance, an AE was defined as any medical complaint except the symptoms/syndrome under study for efficacy. Any abnormal change in laboratory values that was judged by the investigator or the study nurse to be clinically significant was also included. The aforementioned potential AEs were listed on the case report form and regularly screened for at every clinic visit. When the subject filled-in the questionnaire, she or he was required to recall if any of the listed AEs or any symptoms/syndrome under study had occurred between her/his last visit and the current one. Then, both the clinical investigator and the study nurse evaluated the severity and causality of each AE. If either one of them suspected the AE to be related to the TCM therapy, a further causality assessment was conducted for every AE to determine if an adverse drug reaction was involved [19, 20].

We proactively monitored the safety profile of two traditional formulae (*Duhuo Jisheng Tang* and *Suan Zao Ren Tang*) [21, 22] together with a new invented formula named TMN- 1 [23]. These formulae have already been published in evidence-based journals as examples that can be used to demonstrate our strategy of promoting evidence-based medicine as part of TCM.

#### 2.1.1. Prospective Observational Study 1: Short-Term Safety Profiling of the Traditional Chinese Formula, Namely, Suan Zao Ren Tang

*Suan Zao Ren Tang *(*SZRT*) has a long history of use as part of the traditional Chinese pharmacopoeia and was first documented in the classical Chinese text *Jin Gui Yao Lue* (*Essential Prescriptions from the Golden Cabinet*) circa 210 A.D. by Zhong-Jing Zhang [24]. In the classical literature, *SZRT* is said to nourish the blood and calm the nerves eventually bring about a tranquillizing sensation and reduce sleep disturbance. TCM doctors rely on the indications and contraindications mentioned in the thousand-year-old classical Chinese medicine literature when prescribing this TCM therapy. We carry out active safety surveillance for four weeks in order to outline the safety profile of *SZRT* [22].

#### 2.1.2. Prospective Observational Study 2: Short-Term Safety Profiling of a Traditional Chinese Formula Containing Xixin, Namely, Duhuo Jisheng Tang

*Xixin* (*Radix et Rhizoma Asari*), also known as *Saishin* in Japan or *Sesin* in Korea, is widely used in many parts of Asia despite the fact that it contains some minute amounts of aristolochic acid (AA) [25–27]. Although, since 2004, a total of 393 Chinese herbal products containing *Xixin* have been reimbursed under the National Health Insurance (NHI) in Taiwan [28, 29] where the regulations stipulate that AA must be undetectable in the final herbal products [30], the effects of *Xixin*'s plant-derived nephrotoxins and carcinogens on TCM consumers need to be monitored proactively. *Duhuo Jisheng Tang* (*DJT*), a traditional Chinese formula described by the ancient Chinese physician Sun Simiao in 652 AD for the treatment of lower back and knee pain [31] was prescribed to 725,549 patients between 1996 and 2004 in Taiwan. *DJT* has been proposed to be the cause of AA-related nephropathy in a case report [32]. Therefore, a study using active safety surveillance system was conducted to determine whether *DJT* use among patients with osteoarthritis (OA) causes acute nephrotoxicity.

#### 2.1.3. Prospective Observational Study 3: Short-Term Safety Profiling of a New Invented Chinese Formula, Namely, TMN-1

A new formulation called TMN-1, aimed at providing relief to climacteric women with hot flushes, has been created by a TCM expert who has had more than 20 years of practice experience in Taiwan. The product contains a fixed ratio of the three commercially available traditional Chinese medicines, *Jia Wey Shiau Yau San *(*JWSYS*), *Zhi Bo Di Huang Wan *(*ZBDHW*), and *Xiang Sha Liu Jun Zi Tang *(*XSLJT*). All three preparations have traditionally been individually prescribed and are well documented in ancient Chinese medicinal texts (e.g., *JWSYS* in *Prescriptions of the Bureau of Taiping People's Welfare Pharmacy*; *ZBDHW* in *Key to Therapeutics of Children's Diseases*; and *XSLJY* in *Collected Exegesis of Recipes*) [6]. This type of prescription pattern has been neither recommended in the ancient Chinese pharmacopoeias or textbooks nor is it taught as part of the TCM academic program. Hence, there have been concerns regarding herb-herb and/or formula-formula interactions and these cannot be overlooked; as a result, further studies on the safety of such mixed formulae are warranted.

### 2.2. Study Design 2: Analysis of National Databases and/or Established Cohorts

#### 2.2.1. Retrospective Observational Study Targeting the Long-Term Safety Profiles of Traditional Chinese Formulae That Have Been Adulterated with Aristolochic Acid

The datasets for studying the safety of either conventional medicines or TCM therapies can be obtained from any established large health insurance system or from other established cohorts [2]. Many examples are conducted on the reimbursement database established and managed by the National Health Research Institutes (NHRI) for the National Health Insurance (NHI) of Taiwan [33–36]. Out of a total population of 23,400,826 people enrolled within the NHI in Taiwan in 2002, information can be obtained on a random sample of 200,000 or 1,000,000 individuals covering the period of January 1, 1997, to December 31, 2004. We conducted retrospective observational studies based on the reimbursement database of the National Health Insurance to explore the relationship between the use of traditional Chinese formulae that have been adulterated by aristolochic acid in terms of nephrotoxic and carcinogenic risk.

## 3. Results

### 3.1. Prospective Observational Study 1

In all, 61 (91%) of the initial 67 women aged between 45 and 55 years who formed the intention to treat (ITT) group completed the *SZRT* study without any major protocol violation. However, the study raised notable safety issues. A total of 221 AEs were detected/reported during the study period. The most often reported AEs were abdominal distention, diarrhea, cough, headache, and dizziness with the incidence rates of 4.2, 3.6, 3.0, 1.8, and 1.8 per 1000 person-days, respectively, and 1.4, 1.2, 1.0, 0.6, and 0.6 per 1000 person-sachets, respectively (Table 1). Five events were judged to be probably related to the treatment. These consisted of three single events of dizziness/headache/stomach ache and two events of diarrhea during 4-week therapy period. Three participants withdrew from the study due to the AEs. Intolerable side effects resulting in withdrawal included three events of stomach ache, diarrhea, and dizziness. We further carried out periodic evaluation of any unexpected symptoms during the treatment period and found that gastrointestinal discomfort occurred two days after *SZRT* consumption. We were unable to rule out the possibility that consuming *SZRT* might cause a deterioration in some preexisting gastrointestinal and/or neurological symptoms during climacteric period.

### 3.2. Prospective Observational Study 2

Amongst the 71 participants with OA knee, a total of 287 AEs were detected/reported during the study period and were coded according to the Coding Symbols for Thesaurus of Adverse Reaction Terms. The most often reported AEs were rashes, abdominal fullness, coughs, somnolence, muscle cramps, and diarrhea with the incidence rates of 14.5, 12.9, 12.4, 11.9, 10.3, and 10.3 per 1000 person-days, respectively, and 7.5, 6.9, 6.6, 6.3, 5.5, and 5.5 per 1000 person-sachets, respectively (Table 1). These AEs were tolerable and did not have any significant effects on the subjects' daily activities. None of the subjects showed any abnormalities with respect to urinalysis, creatinine, blood urea nitrogen, urinary N-acetyl-glucosaminidase or retinal binding protein [37].

We also conducted an ITT analysis and found that, after four weeks of treatment, 44 of the 68 patients reported no change in the symptom of flaccidity, nine reported improvements, whereas fifteen reported deterioration. Thirty-eight patients reported no change in the symptom of aversion to cold after four weeks of treatment; fourteen reported improvements, while 16 reported deterioration. Contrary to the hypotheses proposed in an ancient text on traditional Chinese medicine, *Qianjin Yaofang*, there seemed to be no consistent improvements with regard to these two symptoms after 4 weeks of treatment.

### 3.3. Prospective Observational Study 3

When we carried out another clinical observation to monitor a new invented formula, TMN-1, it was found that five out of 203 adverse events were judged to be probably related to the treatment; these included three single events of nausea, abdominal pain, and abdominal fullness and two events of diarrhea over the 12-week therapy period (Table 1) [18]. Further analyses showed that TMN-1 treatment resulted in an inferior benefit among postmenopausal women, compared to the benefit obtained among women during the perimenopausal period [23].

In addition, during the study period, there was an isolated occurrence of SAE-acute thrombocytopenia [38]. A 52-year-old woman, who was afflicted with perimenopausal symptoms, including hot flushes, sweating and irregular menstrual cycle, had been taking TMN-1 three times a day without any complaints; however, on the 55th day she discovered gingival bleeding. The investigator at the study site found that the woman had numerous petechiae and ecchymoses over her bilateral shoulders, forearms, palms, and thighs. Her complete blood count showed WBC 3,700/mm^3^, RBC 4,150,000/mm^3^, Hemoglobin 12.6 mg/dL, and platelets 2,000/mm^3^. Her platelet count on the 28th day after medication had been 254,000/mm^3^, and therefore a diagnosis of acute thrombocytopenia was made. She immediately received 12 units of platelet transfusion. Since her platelet count had returned to 99,000/mm^3^ by the following day, she was subsequently discharged after consultation with a haematologist. As a result of a detailed enquiry into the SAE patient's history, we found that there had been concomitant uses of conventional medicines (acetaminophen, mefenamic, and chlorpheniramine) and other folk herbs (*Moringa oleifera* tea) before the occurrence of this SAE. Following our emergency management, the patient is now fully aware that she may be susceptible to acetaminophen, mefenamic, or chlorpheniramine and must be careful to avoid these medications for the rest of her life.

### 3.4. Retrospective Cohort Study

A retrospective follow-up study was conducted using a systematic random sample (200,000 people) from the National Health Insurance reimbursement database over the period 1997–2002. The incidence rates of chronic kidney disease (CKD) and end-stage renal disease (ESRD) were calculated for the whole sample and for those individuals who had ever used TCM product suspected to contain AA. A total of 2,343 new CKD patients and 25,843 new ESRD patients 199,843 persons were included in the final analysis, with an average incidence rate of 1964/106 person-years for CKD and 279/106 person-years for ESRD. After controlling for other risk factors, including age, hypertension, and diabetes, consumption of 60 g of *Mu Tong* or *Fangchi* as part of a herbal supplement was associated with an increased risk of developing both chronic kidney disease and kidney failure [39, 40].

Using the same database, we also conducted a population-based case-control study in Taiwan to examine the association between prescribed Chinese herbal products that contain aristolochic acid and urinary tract cancer. A total of 4,594 patients newly diagnosed with urinary tract cancer (from January 1, 2001, to December 31, 2002) and a random sample of 174,701 control subjects from the entire insured population (from January 1, 1997, to December 31, 2002) were enrolled. After adjustment for age, sex, residence in a township where black foot disease was endemic, and history of chronic urinary tract infection, having been prescribed, more than 60 g of *Mu Tong* and an estimated consumption of more than 150 mg of aristolochic acid were independently associated with an increased risk of urinary tract cancer in the multivariable analyses (*Mu Tong*: at 61–100 g, OR = 1.6, 95% CI = 1.3 to 2.1, and at >200 g, OR = 2.1, 95% CI = 1.3 to 3.4; aristolochic acid: at 151–250 mg, OR = 1.4, 95% CI = 1.1 to 1.8, and at >500 mg, OR = 2.0, 95% CI = 1.4 to 2.9). A statistically significant linear dose-response relationship was observed between the prescribed dose of *Mu Tong* or the estimated cumulative dose of aristolochic acid and the risk of urinary tract cancer, as shown in Table 2 [41]. However, we did not find the association between *Xixin* consumption and an increased risk of CKD, ESRD, and urinary tract cancer [39–41].

## 4. Discussion

Although randomized control trials (RCT) have been recommended as the gold standard to prove the efficacy of TCM therapies, the intensive requirements in terms of resources and time preclude, this approach having widespread implementation [7, 42]. The inclusion and exclusion criteria imposed on subject recruitment during RCT further limit its generalizability. While most ancient books of TCM stipulate the combined use of many herbs under a specific herbal formulae, TCM doctors usually modify or add on prescriptions for patients because of syndrome differentiation [1]; thus, it is extremely difficult, if not impossible, for the prescribed mixtures of herbs to meet the strict requirements of chemistry and quality control of products associated with RCT. Issues may become even more complicated if, as the results of a poorly designed RCT trial, the results contradict the traditional wisdom of practitioners. Thus, there is a tremendous opportunity for observational studies to provide evidence in the area of TCM, beginning with safety profiles and the testing of old indications followed by the proposing of new hypotheses that will allow the initiation of efficacy trials.

Our establishment of an active safety system has shown that the old indications in terms of traditional Chinese formulas need to be regularly updated according to the data generated from evidence-based studies. Although this type of study is observational in nature and may not be suitable for making strong inferences, it provides an opportunity to corroborate previous claim of indications. In fact, the results of the *DJS* study demonstrated that the previously proposed indication of* DJT* with respect to flaccidity of the lower back and knee and aversion to cold, as written on *Qianjin Yaofang* (*Invaluable Prescriptions for Ready Reference*), seemed to be wrong. Although the probable ADRs are tolerable and did not have any significant effects on the subjects' daily activities, we recommend that regulatory agencies need to require pharmaceutical companies to inform their consumers and health care professionals of any of this type of findings, which are based on these scientific evidence, before their *DJT* products enter the market. The above empirical experiences have shown that active safety surveillance system is feasible for the detection of adverse effects and the validation of old indications of TCM as long as close cooperation among investigators, study nurses, and patients is able to be established. Similarly, we believe that TCM doctors now will be alert to the possibility that prescribing *SZRT* may cause severe gastrointestinal adverse effects or dizziness based on our observational results. As safety and/or adverse event evidence accumulates, these should influence the prescription behavior of TCM doctors. Traditional use, which refers to documentary evidence that related to the recorder use of a traditional Chinese herbs/formula has been used over three or more generations of recorded use for a specific health related or medicinal purpose, is one type of information which that can be used to support claims on TCM. The present findings are another type of evidence indicating the urgent need for changes of the labels of popular *SZRT* products in order to reflect these new concerns. We therefore believe that there should be updating of the old contraindication of *SZRT* based on the relative strengths of the evidence-based claims and their likely impact on consumers. Furthermore, this type of active safety system enables us to exclude without doubt the possibility that TMN-1 alone was the cause of a serious case of thrombocytopenia. Hence, the aforementioned observational studies are able to provide more and better evidence for rational TCM use and in the process improve patient care regardless of whether they have a positive or a negative result.

Of all analytical studies, prospective cohort studies best meet the criteria to clearly demonstrate an appropriate temporal sequence between treatment and outcome. No acute renal tubular damage was observed after receiving four weeks of *DJS* (possibly a total of 1.34–2.01 *μ*g of AAI) during the study period [43]. Possibly, as compared to the reported case of the AA-related nephropathy that involved a patient ingesting 400 g of *DJT* for over four months, the ingestion levels of AA in our observational study were very low. However, it is worth noting in this context that this type of study design may not always be feasible due to its resources requirements in terms of a large sample size that will allow the detection of a low incidence as well as the need for a long periodic followup in order to assess correctly aristolochic acid exposure. Therefore, we must learn from the case of AA nephropathy that was first detected by a Belgian physician [17] rather than from countries with a long history of taking Chinese herbs, such as China, Japan, Korea, or Taiwan. Although traditional Chinese therapies have been used for many thousands of years, the data generated from our observational studies show that they cannot be guaranteed to be totally safe [39–41, 44, 45]. Furthermore, the herbology theory of traditional Chinese medicine, in which different herbs are mixed together to counteract their toxicity, may not always be correct. Thus, a retrospective observational study should be useful when exploring serious adverse effects, such as chronic renal failure and/or urothelial cancer associated with exposure to aristolochic acid containing Chinese formulae. As the sensitivity of such studies might be reduced by limited sample sizes and misclassification errors resulting from consumption of herbs outside of prescriptions, future long-term active surveillance of *Xixin* (potential containing aristolochic acid) consumers or users is still needed so that a careful watch on the safety of current regulatory policy is maintained.

In conclusion, prospective or retrospective observational studies have specific advantages when investigating safety of and adverse effects of TCM therapies and possibly other alternative/complementary therapies. The usefulness of such studies to prove efficacy is limited because bias in patient selection may occur. The evidence we obtain from such studies, however, can provide highly useful data on the safety profile of daily TCM practice, which will also serve as prior information when designing randomized control trials. In general, it is ethically and financially more feasible to carry out observational studies to generate data and/or information on the adverse effects of existing TCM therapies that to tackle a risk/benefit evaluation [46–48]. Moreover, the more information we obtain about the safety and adverse events of TCM therapies and alternative/complementary medicines, the greater is the likelihood that physicians trained in conventional medicine will be encouraged to use such medicines rationally. This will possibly help to bridge the gaps between these two fields [49].

## Figures and Tables

**Table 1:** Active surveillance system for herbs safety: top 20 adverse events detected by the panel of investigators and study nurses during the study period.

Adverse events	Risk*					
	*Suan Zao Ren Tang *		*Duhuo Jisheng Tang *		TMN-1	
	/10^3^ person-days^a^	/10^3^ person-sachets^a^	/10^3^ person-days^b^	/10^3^ person-sachets^b^	/10^3^ person-days^c^	/10^3^ person-sachets^c^
Body as a whole						
Abdominal pain	1.8	0.6	8.3	4.4	1.8	0.6
Abdominal fullness	4.2	1.4	12.9	6.9	1.7	0.6
Dizziness	1.8	0.6				
Chest pain	0.6	0.2	3.1	1.7	0.3	0.1
Fever	0.6	0.2	1.0	0.2	0.2	0.1
Somnolence	—	—	11.9	6.3	—	—
Musculoskeletal system						
Muscle cramps	—	—	10.3	5.5	0.1	0.1
Headache	1.8	0.6	4.6	2.5	—	—
Respiratory system						
Cough	3.0	1.0	12.4	6.6	2.6	0.9
Rhinitis	1.8	0.6	8.3	4.4	1.9	0.7
Pharyngitis	1.8	0.6	8.3	4.4	2.5	0.9
Digestive system						
Diarrhea	3.6	1.2	10.3	5.5	1.6	0.6
Nausea	1.2	0.4	3.1	1.7	0.8	0.3
Vomit	0.6	0.2	0.5	0.3	0.3	0.1
Oral ulcer	—	—	1.5	0.8	0.2	0.1
Stomachache	0.6	0.2	—	—	0.5	0.2
Skin and appendages						
Urticaria	—	—	0.5	0.3	0.1	0.0
Pruritus	0.6	0.2	—	—	1.6	0.6
Skin discolor	—	—	0.5	0.3	0.3	0.1
Rash	0.6	0.2	14.5	7.7	0.8	0.3

*Number of cases was used as the numerator for calculation of risk.

^a^The denominators for the calculation of the risks were 1,691 person-days and 5,122 person-sachets.

^b^The denominators for the calculation of the risks were 1,936 person-days and 3,633 person-sachets.

^c^The denominators for calculation of risks were 10,133 person-days and 28,744 person-sachets.

**Table:** (a)

Cox regression model for chronic kidney disease^a^			
Study Chinese herb	No. of cases	HR	95% CI
Mu-Tong			
0 g	1,979	1.0	
1–30 g	248	1.0	0.8–1.1
31–60 g	63	1.3	1.03–1.8
61–100 g	27	1.4	0.96–2.1
101–200 g	22	1.7	1.1–2.6
>200 g	4	0.7	0.3–1.9
Fangchi			
0 g	1,875	1.0	
1–30 g	389	1.0	0.9–1.2
31–60 g	42	1.3	0.98–1.9
61–100 g	18	1.8	1.1–2.8
101–200 g	10	1.4	0.8–2.7
>200 g	9	2.2	1.1–4.2

**Table:** (b)

Multiple logistic regression model for kidney failure^b^			
Study Chinese herb	No. of cases/controls	Adjusted OR	95% CI
Mu-Tong			
0 g	22,188/157,939	1.0	
1–30 g	2,542/20,122	1.12	0.86–1.47
31–60 g	492/3,729	1.16	0.83–1.62
61–100 g	226/1,569	1.47	1.01–2.14
101–200 g	209/1,054	2.14	1.47–3.11
>200 g	186/438	5.82	3.89–8.71
Fangchi			
0 g	21,985/157,543	1.0	
1–30 g	3,145/24,868	0.68	0.58–0.78
31–60 g	362/1,528	1.14	0.91–1.44
61–100 g	169/492	1.60	1.20–2.14
101–200 g	116/295	1.62	1.17–2.23
>200 g	66/125	1.94	1.29–2.92

**Table:** (c)

Multiple logistic regression model for urinary tract cancer^c^			
Study Chinese herb	No. of cases/controls	Adjusted OR	95% CI
Mu-Tong			
0 g	3,987/149,464	1.0	
1–60 g	489/22,354	1.0	0.9–1.2
61–100 g	50/1,485	1.6	1.3–2.1
101–200 g	46/1003	2.0	1.4–2.7
>200 g	22/395	2.1	1.3–3.4
Fangchi			
0 g	3,927/150,456	1.0	
1–60 g	623/23,456	0.9	0.8–1.0
61–100 g	15/427	0.7	0.4–1.2
>100 g	29/362	1.3	0.9–2.0
Estimated cumulative dose of aristolochic acid, in mg			
0	3,274/121,820	1.0	
1–150	1151/48,869	1.0	0.96–1.1
151–250	69/2,032	1.4	1.1–1.8
251–500	64/1,403	1.6	1.2–2.1
>500	36/577	2.0	1.4–2.9

^a^Hazards ratios (HR) and 95% confidence intervals (CI) obtained from Cox proportional hazards regression models with all variables (sex, age, hypertension, diabetes, cumulative dosage of nonsteroidal antiinflammatory drugs, Mu Xiang, Mu-Tong, and Fangchi) fitted simultaneously.

^b^Multivariable odds ratios were adjusted for potential confounders (sex, age, hypertension, diabetes, chronic hepatitis, chronic urinary tract infection, chronic neuralgia, musculoskeletal disease, cumulative dosage of nonsteroidal antiinflammatory drugs, Mu Xiang, Mu-Tong, and Fangchi) fitted simultaneously.

^c^Multivariable odds ratios were adjusted for potential confounders (sex, age, hypertension, diabetes, residence in township where black foot disease was endemic, chronic urinary tract infection, Mu Xiang, Mu-Tong, and Fangchi) fitted simultaneously.
